# Clinic of Prof. Garretson at the Medico-Chirurgical College, Philadelphia

**Published:** 1885-05

**Authors:** 


### CLINIC OF PROFESSOR GARRETSON AT THE MEDICO-CHIRURGICAL COLLEGE, PHILADELPHIA.

### SERVICE IN ORAL SURGERY.

REPORTED FOR THE INDEPENDENT PRACTITIONER.

### CASE I.—HYPERTROPHY OF THE TONSILS.

The patient presented with a marked enlargement of the tonsil of the left side, the excessive growth causing much interference with speech, and impairing the sense of hearing by pressure upon the Eustachian tube. The condition being of several years' duration, and causing much inconvenience, request was made for removal of the gland, either by cauterization or excision. The professor preferring the cautery to the more severe operation, made use of London paste as the medium for treatment. This preparation consists of equal parts of quick lime and caustic soda, mixed into a mass by the addition of a few drops of absolute alcohol. Placing the patient in such a position as to get full advantage of the light, the tongue was held down with a depressor, and a small quantity of the paste applied to the parts, upon the cup of an ordinary director, and held in contact about one minute. Upon its removal the enlarged surface was seen to assume a dark-red color, quickly followed by a deep purple, a few hours later the eschar taking on a yellowish-white appearance. The remark was made that anyone, with ordinary precaution, could apply so simple a remedy, care only being required to mix the paste to proper consistency. If too thin, it will run over surrounding tissue and cause unnecessary destruction; if too thick, it will not adhere to the parts intended to be removed. Should such an accident occur, the fauces should be immediately gargled with water. The case was cured by three similar applications to the one described, repeated at intervals of four days, the pain accompanying the treatment being inconsiderable.

### CASE II.—EXSECTION OF THE AURICULO TEMPORAL NERVE.

This was a case in which the patient had suffered some years with neuralgia in the region traversed by this nerve, for which she had received varied remedies of a sedative kind, amongst others local applications of chloroform, belladonna, aconite, opium, hypodermic injections, etc., in addition to constitutional treatment, but had only been temporarily relieved. The success attending operations for facial neuralgia in this clinic prompted her to come to it, in the hope of obtaining more permanent benefit. Causes of neuralgia of this class are not unfrequently so obscure that any treatment resorted to is likely to be experimental at best, no diagnosis being more difficult than that connected with what is ordinarily termed neuralgia. The knowledge that successful results have been obtained in similar cases, prompted the performance of an operation as the only remaining hope of ridding the patient of the almost constant pain to which she had been subjected. Simple division of a nerve is not sufficient, but excision of a portion is necessary, otherwise it will reunite, and the continuity being re-established, the operation certainly fails. The procedure consisted in first marking out the position of the superficial temporal artery, behind which the nerve ascends over the base of the zygoma. An incision upwards, about one inch in length, was then made through the integuments, the artery being cut and ligated. (Ligation of the artery is not a necessity, but in this case was practiced because of the great congestive condition exhibited about the parts.) The nerve was found in position, and a portion excised. The parts were next drawn together, two stitches inserted, and compresses saturated in phenol-sodique placed at each side of the wound. An overlying external pad completed the dressing.

### CASE III.—EPULIC TUMOR.

To state that a growth is an epulic tumor gives no clue as to diagnosis, the assertion merely indicating position, the name being given to all abnormal growths upon the gum. The patient introduced had an extensive tumor, situated in such a position as to necessitate the removal of the whole of the alveolar process of the left superior maxilla, from the central incisor tooth to the tuberosity, the intervening teeth being in position. The growth was of an indurated nature, arising mainly from the periosteum, osseous trabeculæ passing from the bone beneath. In the early stages of such tumors the mucous membrane is pushed outwards, causing small prominences of a rounded shape to appear behind, in front, or between the teeth, eventually assuming extensive proportions. The operation consisted in first extracting the central, lateral, and bicuspid teeth, these latter being involved in the growth, then with a circular saw, revolved by the surgical engine probably eight thousand times a minute, the whole of the involved portion of the bone, consisting of the alveolar process and a portion of the floor of the antrum, was cut away, the time occupied being but a few minutes. An operation of this character, previous to the adaptation of the dental engine to surgical purposes, was exceedingly complex, necessitating the making of a cut from the angle of the mouth up the side of the face, and the removal of the diseased parts by the aid of hand, saw and bone forceps, uncertainty prevailing as to whether or not all the involved portions were cut away. Operating with the engine and saw in the manner described, the professor was enabled to remove only such parts as were connected with the growth, and to say emphatically that it would not recur, neither would any disfigurement result. During the operation the posterior, or descending palatine artery, which supplies the gum tissue of the upper jaw, hard palate and alveoli, was cut, the hemorrhage being controlled by the insertion of a plug into the canal. After-treatment consisted in syringing out the debris, and washing the parts with dilute phenol-sodique. The patient rapidly recovered from the effects of the operation, and was discharged from the hospital ten days afterwards.

